# Adolescent sexual and reproductive health and rights: a stock-taking and call-to-action on the 25th anniversary of the international conference on population and development

**DOI:** 10.1080/26410397.2019.1676006

**Published:** 2019-11-08

**Authors:** Venkatraman Chandra-Mouli, Marina Plesons, Arup Barua, Anshu Mohan, Meheret Melles-Brewer, Danielle Engel

**Affiliations:** a Scientist, Adolescent Sexual and Reproductive Health, World Health Organization Department of Reproductive Health and Research/Human Reproduction Programme, Geneva, Switzerland.; b Consultant, Adolescent Sexual and Reproductive Health, World Health Organization Department of Reproductive Health and Research/Human Reproduction Programme, Geneva, Switzerland; c Postgraduate Student, Central European University, Budapest, Hungary; d Senior Technical Officer, Country Engagement, Partnership for Maternal, Newborn and Child Health, Geneva, Switzerland; e Technical Officer, Adolescent Health and Wellbeing, Partnership for Maternal, Newborn and Child Health Geneva, Switzerland; f Technical Specialist, Adolescents and Youth, United Nations Population Fund, UNFPA, New York, USA

## Introduction

In 1994, the International Conference on Population and Development (ICPD) put forward a groundbreaking call to place adolescent^1^ sexual and reproductive health and rights (ASRHR) on the agenda. Specifically, it called for countries to meet adolescents’ educational and service needs to enable them to deal in a positive and responsible way with their sexuality.

Much has changed in the lives of adolescents in the last 25 years. More adolescents are alive than ever before, they are more likely to be in school, and they are more likely (than children and adults) to be digitally connected. Although they are less likely than before to live in absolute poverty, they still struggle with employment upon entering adulthood and many are affected by humanitarian crises and face an uncertain future due to climate crises.

Alongside these changes, there has been significant – albeit uneven – progress in their sexual and reproductive health. Girls are less likely to be married and bear children – within or outside marriage – before age 18, but are still more likely than adults to have unintended pregnancies and unsafe abortions. Adolescents today are less likely to get HIV than they were in the past, but are less likely than children or adults to obtain HIV testing and care. Finally, levels of sexual abuse in boys and especially girls under 18 and of sexual violence in girls aged 15–19 remain shockingly high.^1,2^

## How have political, programmatic, and social responses to ASRHR evolved over the last 25 years, and what is the unfinished agenda?

*Firstly, ASRHR is higher on the agenda and has more financial investment*.

Selected aspects of adolescent health were included in the Millennium Development Goals (MDGs). However, the critical investments needed to ensure adolescents’ successful transitions into adulthood were largely neglected in the face of other pressing issues.^3^ The consensus from the MDG experience – that adolescents should not be left behind – resulted in their strong presence in the Sustainable Development Goals.

Concerted advocacy by many groups, including youth champions, on the demographic, health, economic, and human rights rationales for investment in adolescent health contributed to placing ASRHR on global, regional, and national agendas. However, despite consensus on the need to address some areas of ASRHR such as child marriage, other areas such as comprehensive sexuality education (CSE) still face resistance.

While adolescent health gets only a modest portion of global development funding, investment in specific areas of ASRHR such as HIV, child marriage, and contraception has grown steadily.^4^ However, the available financing is still inadequate, fragmented, and primarily from external sources, hindering progress on ASRHR, especially on geographic coverage of interventions.

*Secondly, there is much more data and evidence on ASRHR, which have fed into norms/standards to guide policies and programmes*.

Evidence has grown on the heterogeneity of adolescents, as well as on the nature and scale of ASRHR problems; their causes in different contexts; their consequences to individuals, families, and communities; and the effectiveness of interventions to address them. However, there are still important gaps, for example on very young adolescents and on costs of interventions.^1^

Growing consensus on effective approaches to address ASRHR and the development of related normative guidance have provided strategic direction for decision-makers and encouraged investment. However, evidence-based guidelines still do not always reach/influence decision-makers.

*Thirdly, a growing number of countries have enabling laws/policies, government-led programmes and positive social responses for ASRHR*.

Many countries have passed laws to end harmful traditional practices such as child marriage. Alongside efforts to strengthen these laws – for example, to avoid exceptions – efforts are underway to strengthen their application. However, restrictions remain in many countries, such as providing contraceptives to unmarried adolescents.

NGOs were among the first to respond to the needs and problems of adolescents, and continue to be innovators, implementers, and watchdogs. Increasingly, governments have developed policies, strategies, and programmes. In a small but growing number of countries, they have demonstrated that tangible results are possible through good science, leadership, management, and persistence.^5^ In many places though, programmes are poorly designed, implemented, managed, and measured, meaning that systems are still largely not geared to adolescents.

While there is social support for some areas of ASRHR in many countries, there are areas such as safe abortion that still encounter substantial resistance. The bedrock of this is a persistent denial of and discomfort with adolescent sexuality. As a result, efforts to promote ASRHR face well-financed and organised opposition, which can stall and even reverse progress.^6^ Finally, robust grassroots movements, including some led by young people, have become increasingly active and influential in demanding an equal place at the table.

## What could we do to build on the progress made and lessons learned?

*Firstly, we must advocate to keep ASRHR on the agenda, meaningfully involve adolescents, and hold stakeholders accountable*.

We must press for ASRHR to stay on the agenda and ensure that it is not sidelined in important initiatives such as Universal Health Coverage. Where there is inadequate readiness to address ASRHR, we must press for action with data and compelling stories about the consequences of neglecting adolescents, as well as examples of where ASRHR has been addressed effectively. Even where there is nominal support for ASRHR, we must address the denial that adolescents – including those with disabilities – are sexual beings, press for normalisation of discourse on sex – including pleasure – and fight the stigmatisation/criminalisation of those with diverse gender and sexual orientations.

We must ensure that adolescents are meaningfully involved by guaranteeing them a place at the table, supporting them to carry out clearly defined roles, and acknowledging and compensating their contributions.^7^

Finally, we must hold stakeholders who have committed to investing in and/or acting on ASRHR accountable by tracking and reporting on their contributions.^8^

*Secondly, we must fill crucial data and evidence gaps, while working to put available data and evidence to full use*.

We must invest in filling the data and evidence gaps that contribute to misinformed policies/strategies, such as those on the contraceptive needs and preferences of unmarried adolescents. In filling such gaps, we must engage young people and prioritise implementation research to learn what it takes – and costs – to deliver effective interventions at scale with quality and equity in different contexts.^9^

Lastly, we must reach decision-makers with available data and evidence and build their capacity and willingness for well-informed decision-making.

*Thirdly, we need to strengthen laws/policies and programmes on ASRHR with a dogged focus on the last mile and on those who might be left behind*.

In countries with enabling environments, we must ensure that frontline workers are aware of existing laws/policies and of their obligations to apply them; and that adolescents are aware of their rights and entitlements. We must also strengthen governance systems to apply and monitor these laws/policies. Where legal/policy barriers remain, we must identify those that are most critical and work to change them. In all countries, we must make full use of enabling laws/policies in designing strategies, and build on the learnings of the last 25 years: for example, that adolescents are a heterogeneous group with diverse needs; that we must make full use of critical inflection points in adolescents’ lives; that changing deeply ingrained gender norms requires sustained interventions that operate from local to national levels; that different sectors can be nudged to work together with incentives and obligations; and that building health worker performance requires more than one-off training.

Sound strategies provide the basis for scale-up plans that specify the interventions and spell out *who* we want to reach, *where, when,* and *how*. In choosing delivery platforms, we should leverage emerging opportunities such as growing secondary school attendance and mobile phone access. We must also emulate the “last mile” focus of the pharmaceutical supply chain management field.^10^ For example, while we continue to develop CSE curriculum and train teachers, we must focus on ensuring that every adolescent is provided with accurate/appropriate information and engaged in discussions in a safe environment. These efforts must be combined with work to build community support, and to anticipate and respond to opposition.

Finally, ASRHR policies and programmes of the 2020s and beyond must consider wider influences such as demographic changes, displacement and conflict, and climate change. These changes affect all aspects of health and development and call for whole-of-government approaches, firmly grounded in well-coordinated inter-country efforts.

## Footnotes

1 WHO defines adolescents as people between the ages of 10–19 years.

## References

[1] WHO WHO recommendations on adolescent sexual and reproductive health and rights. WHO, Geneva 2018.

[2] Azzopardi SP, Heaps JCS, Francis LK et al. Progress in adolescent health and wellbeing: tracking 12 headline indicators for 195 countries and territories, 1990-2016. Lancet 2019;393:1101-1118. 10.1016/S0140-6736(18)32427-9 PMC6429986 30876706

[3] Chandra-Mouli V. The world has much to do to achieve the International Conference on Population and Development’s adolescent-health objectives. Sex Reproductive Healthc. 2015;6:1-2. doi: 10.1016/j.srhc.2014.12.001 25637416

[4] Li Z, Li M, Patton GZ, et al. Global development assistance for adolescent health from 2003-2015. JAMA Netw Open. 2018;1(4):10.1001/jamanetworkopen.2018.1072. PMC6324521 30646101

[5] Chandra-Mouli V, Plesons M, Hadley A et al. Lessons learned from national government-led programmes to reduce teenage pregnancy in Chile, England and Ethiopia. Early Child Matters, 2019;128:50-56.

[6] Darsa Understanding and overcoming backlash of girls’ exercise of agency in India. Darsa, India, 2019. Available from: https://www.dasra.org/resource/action-reaction-understanding-and-overcoming-backlash-against-girls-exercise-of-agency-in-india

[7] PMNCH Global consensus statement on meaningful adolescent and youth engagement. PMCH, Geneva. 2018. Available from: https://www.who.int/pmnch/mye-statement.pdf

[8] Independent Accountability Panel Transformative accountability for adolescents: Accountability for the health and human rights of women, children and adolescents in the 2030 agenda. WHO, Geneva 2017.

[9] MAMTA Health Institute for Mother and Child. Report of Technical Consultation: Adolescent Health and Implementation Science: Achievements, Challenges and Prospects (2016). MAMTA, New Delhi. 2016. Available from: http://mamta-himc.org/pdf/Technical-consultation-Report-2016.pdf

[10] Price Water Coopers Improving Global Health Outcomes Through Last Mile Logistics Special Focus Forum – Global Health and Humanitarian Supply Chains. Price Water Coopers. Washington DC, 2017. Available from: https://www.pwc.com/us/en/health-industries/health-services/assets/global-health-outcomes-through-last-mile-logistics.pdf

